# Is the kinetome conserved?

**DOI:** 10.15252/msb.202110782

**Published:** 2022-02-21

**Authors:** Bernhard O Palsson, James T Yurkovich

**Affiliations:** ^1^ Department of Bioengineering University of California San Diego La Jolla CA USA

**Keywords:** Computational Biology, Metabolism

## Abstract

Computational biologists have labored for decades to produce kinetic models to mechanistically explain complex metabolic phenomena. The estimation of numerical values for the large number of kinetic parameters required for constructing large‐scale models has been a major challenge. This collection of kinetic constants has recently been termed the *kinetome* (Nilsson et al, 2017). In this Commentary, we discuss the recent advances in the field that suggest that the kinetome may be more conserved than expected. A conserved kinetome will accelerate the development of future kinetic models of integrated cellular functions and expand their scope and usability in many fields of biology and biomedicine.

## Graphical abstract

This Commentary highlights recent discoveries suggesting that the kinetome (a collection of kinetic parameters) can be conserved and discusses how a conserved kinetome will accelerate the development of future kinetic models.

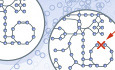

## Genome‐scale models

Once whole‐genome sequences became available in the mid‐1990s, the reconstruction of metabolic networks reached the genome‐scale. Over the past two decades, genome‐scale metabolic models (GEMs) built from these network reconstructions have contributed to our understanding of systems biology of metabolism, with applications ranging from metabolic engineering to bacterial evolution to infectious disease. Genetic variation can now be incorporated into genome‐scale models of metabolism, transcription, translation, proteostasis, and cellular stresses. GEMs are often simulated using flux balance analysis that requires a minimal number of parameters estimated from empirical data.

Kinetic models, on the other hand, have traditionally been limited by the need for extensive parameterization. If the kinetome can be estimated from high‐throughput data, then we can develop large‐scale—even genome‐scale—kinetic models. Since GEMs have a direct genetic basis, a new generation of kinetic models can be directly rooted in protein structures and sequence variation. The kinetome of such models would be large, requiring the estimation of many unmeasured parameters. However, if segments of the kinetome are conserved, then parameterization will be simplified. Reference kinetomes can be estimated for well‐characterized strains and applied to less well‐known strains.

## Estimating the kinetome using omic data

**Figure 1:**
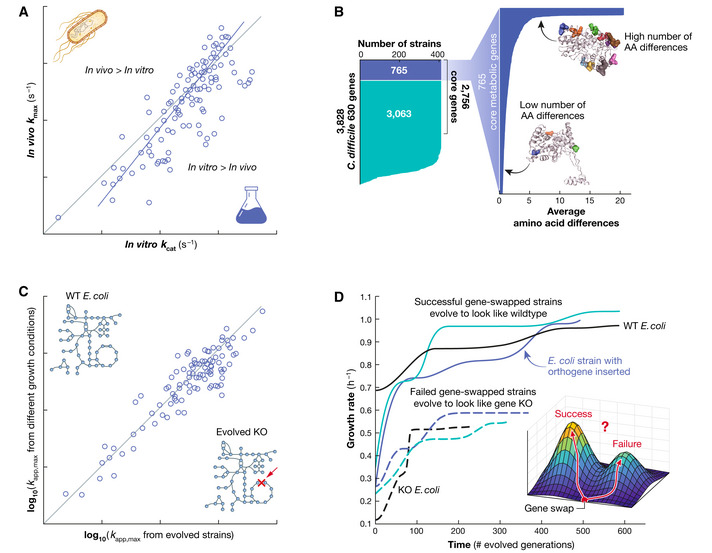
The numerical values of turnover rates determine how much protein is needed to achieve a homeostatic state Recent studies have explored the numerical values of turnover rates, allelic variation, and the importance of mechanisms that influence enzyme abundance. A number of recent advances show that the $k_{\mathrm{cat}}^{in\;vivo}$ is a relatively constant parameter (panels A and C), and *e_i_* varies *in vivo* and is achieved through a variety of regulatory mechanisms (panels B and D). (A) A comparison of *in vivo* and *in vitro* kinetic parameters through the use of omic data shows reasonable association in *E. coli* (*r* ^2^ = 0.62); adapted from Davidi *et al* (2016). (B) Core metabolic genes identified in 400 strains of *C. difficile* shown in blue. The pull‐out horizontal bar chart shows the variation in these metabolic alleles as measured by the average number of amino acid substitutions across wild‐type strains. Most metabolic genes have conserved alleles, suggesting that the kinetic parameters for these enzymes are also conserved. Adapted from Norsigian *et al* (2020). (C) Comparing numerical estimates *k_app,max_* obtained from evolved enzyme knock‐out *E. coli* strains and *k_app,max_* from growth conditions shows a strong association (*r* ^2^ = 0.9). The *in vivo* turnover rates were estimated in the same metabolic specialist grown on the substrate used for the evolution and an alternative substrate. Adapted from Heckmann *et al* (2020). (D) Allele swaps of the coding region of a metabolic gene in wild‐type *E. coli* with an orthogene from another species. The strain is then evolved and either the orthogene allele adapts function after the evolution and the strain gets close to the wild‐type growth rate (solid lines), or the allele fails to adapt and after evolution the strain shows a similar growth as the evolved knock‐out strain (dashed lines). In the former case, most adaptive mutations are related to enzyme abundance and not structural mutations that would change the kinetic parameters of the enzyme. Adapted from Sandberg *et al* (2020).

As omic technologies advanced, it was recognized that multi‐omic data combined with GEMs could lead to the estimation of a large set of enzyme turnover rates, the most important kinetic parameters in the kinetome. Indeed, *in vivo* turnover rates of bacterial enzymes have been characterized using ratios of proteomic and fluxomic data (Davidi *et al*, 2016). This landmark study showed that *in vitro* enzyme assays concur with maximal *in vivo* rates for many enzymes, and that *in vivo* estimated parameters could be used to fill in some of the scarcity in the parameterization of large‐scale kinetic models (Fig 1A).

## Enzyme turnover rates are largely conserved

A few recent studies have suggested that the kinetome is more conserved than previously thought. The abundance of available whole‐genome sequences allows for large‐scale allelic comparison across metabolic genes, and the initial analysis of such data shows that most metabolic genes have a low amino acid substitution rate (Norsigian *et al*, 2020) (Fig 1B). Thus, only a small number of metabolic genes seem to face selection pressures, suggesting that their estimated enzyme turnover rates may have broad applicability.

This potential broad applicability of estimated turnover rates has been further supported by two recent adaptive laboratory evolution (ALE) studies. ALE allows for the generation of strains that have adapted to high growth rates following the deletion of genes that encode specific metabolic enzymes. This approach results in the generation of “metabolic specialist” strains whose pathway usage has been rewired by ALE following the loss of a key metabolic enzyme. A large study resulted in an estimation of turnover rates for the same enzyme in multiple metabolic specialists (Fig 1C). These estimates were consistent among the specialists and with the wild type. Consistently, only relatively few structural mutations were identified, but regulatory mechanisms altered the abundance of metabolic enzymes, resulting in the required alteration of metabolic fluxes (McCloskey *et al*, 2018).

A second ALE study swapped glycolytic genes in *E. coli* with orthogenes from a diverse range of other species, from hyperthermophilic archaea to humans (Sandberg *et al*, 2020). Following ALE, many *E. coli* lineages adapted to use the orthogenes to replace their own. Adaptive mutations were rarely found in orthogene coding sequences, with the majority of mutations falling within regulatory regions that altered enzyme expression levels. These two ALE studies suggest that optimal flux levels *in vivo* are more often impacted by the adjustment of an enzyme’s abundance rather than an alteration to its turnover rate (Fig 1D). Again, these results suggest that the kinetome may exhibit a notable degree of conservation.

## The implications of a conserved kinetome

The reason for the relatively small sequence variance in metabolic alleles—and thus the numerical values of kinetic parameters—is not clear. One possibility is that key enzymes have evolved to a relatively high catalytic efficiency, and that the regulation of metabolic flux is achieved through altering protein abundance between different conditions. In other words, the cellular components remain the same, but their relative abundance is adjusted. This observation has two significant implications. First, large‐scale kinetic models will be more easily constructed for related strains and species due to the broad applicability of estimated numerical values for kinetic parameters. Second, a conserved kinetome means that the enzyme abundances become a key issue in determining *in vivo* fluxes. Thus, models that explicitly compute protein abundances will grow in importance as they directly assess the phenotypic consequences of proteome allocation (Chen & Nielsen, 2019).

Models that constrain metabolic fluxes with protein abundances have been developed. These models either compute the composition of the abundances of the enzymes (so‐called metabolism and expression (ME) models), or they specify the enzyme abundances based on measurement (enzyme‐constrained models). Ultimately, these models enable the study of the principles that underlie how the limited number of protein molecules that a cell can carry are optimally allocated to metabolic enzymes that have an inherent set of kinetic constants (Chen & Nielsen, 2019).

Turnover rates and enzyme abundances will both become important considerations as kinetic models grow larger, thus tightly integrating molecular and systems biology. Such integration places component properties in the context of the whole, and the principles for overall functionality become based on optimization—an evolutionary principle. The computation of optimal proteome composition may become known as “proteometrics” in analogy to econometrics.

## A new generation of genome‐scale kinetic models

So how will a conserved kinetome affect the construction of future models? The incorporation of kinetic information and other multi‐omic data into GEMs represents an advance in computational biology, expanding the scope and utility of models that describe kinetic effects. GEMs have continued to integrate more and more multi‐omic data, with some of the most recent models predicting whole‐body metabolism in humans (Thiele *et al*, 2020).

Kinetic parameterization is a challenge for the generation of models for personalized medicine applications due to both the large number of parameters required and the inherent individual variation. This challenge would in part be resolved if conserved features of the human kinetome were identified and the variable parts could be traced to sequence variation. While the metabolic kinetome may be largely conserved, key mutations—such as those causing G6PDH deficiency—are known to affect cellular function. The impact of sequence variation on the kinetome has been examined in the human red blood cell (RBC), providing insight into whether different omic data types can be used together to infer kinetic parameter values.

The RBC is the most abundant cell in the human body, thus representing an important starting point for the development of personalized medicine in human systems. In a recent study, personalized RBC kinetic models were constructed through parameterization based on metabolomic, fluxomic, and genotyping data (Bordbar *et al*, 2015). Remarkably, this first set of personalized RBC models showed that individual differences occur on physiologically relevant timescales of erythrocyte circulation. They also predicted personalized pharmacodynamic responses and identified individuals at risk for a ribavirin‐induced anemic side effect, providing a mechanistic explanation for how genetic variation (inosine triphosphatase deficiency) may protect against this drug side effect. Since the proteome was not determined in this study, enzyme abundances were standardized across all individual models. Thus, the turnover rates and enzyme abundances were not independent parameters—yet their combination was notably shown to have a stronger correlation with genotype than metabolomics data. In the future, *ex vivo* erythropoietic models might be used in conjunction with gene editing of the adult hematopoietic stem cells to engineer specialist RBCs.

## A path forward

If we can scale up and validate such personalized models for other human cell types, we could make progress toward the development of personalized wellness and disease models in humans. Progress is being made in this area, with one study reporting the use of transcriptomic data (as a proxy for proteomic data) from individual tumor samples to constrain maximum flux rates for cancer patients (Lewis *et al*, 2021). Currently, vast amounts of multi‐omic data are required to capture all relevant biological functions and properly parameterize personalized kinetic models. However, if the kinetome proves to be conserved for different human cell types, then only key variants (like those that cause G6PDH deficiency) will need to be measured for models to be accurately parameterized.

Parameterized GEMs are likely to have many uses in the life sciences. In particular, GEMs are likely to be used for the purposes of designing new genomes and to simulate their phenotypes *a priori*. We are faced with challenges that stem from our incomplete understanding of the genome, with many uncharacterized genes still present in even the most well‐studied model organisms. Furthermore, there are challenges associated with the complex regulatory networks that govern cellular functions, and mechanisms that will need to be better understood if they are to be explicitly incorporated into computational models. Yet if these challenges can be overcome, the resulting models could lead to the design of novel genomes that can be constructed in a laboratory and function as self‐sustaining organisms—an application that would represent a key milestone in the history of biology.

Genome‐scale kinetic models rooted in protein structures and sequence variation offer the promise of bringing us closer to really understanding the genotype–phenotype relationship to a point where we can design and build genotypes that have desired phenotypes. Defining the variable and conserved part of a kinetome will lead to computational models that can help with designing new genomes by assisting genome designers in identifying the sequence variation with phenotypic consequences.

## Author contributions

**Bernhard O Palsson:** Conceptualization; Funding acquisition; Writing—original draft; Writing—review & editing. **James T Yurkovich:** Conceptualization; Writing—original draft; Writing—review & editing.

**Box 1: A shift from kinetic parameters to enzyme concentrations and proteome allocation**

Maximum flux catalyzed by an enzyme is determined by a product of the turnover rate (reactions/protein molecule/time that is on average approximately 10s of reactions per second) and protein concentration (protein molecules per unit volume) through the well‐known equation, *V_m_* = *k_cat_* × *e_tot_*, used to describe enzyme kinetics *in vitro*.

The *in vivo* flux (*v_i_*), determined via fluxomics, is given by *v_i_* = *k_app,i_* × *e_i_*, where *k_app,i_* is the apparent catalytic rate for enzyme *i in vivo*, and *e_i_* is the *in vivo* enzyme concentration as determined by proteomic measurements. The term *k_app,max_* (the maximum *k_app_* observed across multiple conditions) is a proxy for $k_{\mathrm{cat}}^{in\;vivo}$ and can be used in simulation models. When working with a large number of enzymes, *e_i_* becomes constrained by limited proteome size ($\sum_{i=1}^{n}e_{i}$), and thus proteome allocation becomes important (Nilsson *et al*, 2017). Enzyme‐limited models offer new challenges, such as determining what fraction of synthesized enzyme molecules are actively involved in catalysis. Furthermore, thousands of reactions have to be balanced to achieve a stable function of a metabolic network. Thus, the focus of construction of large‐scale kinetic models shifts from kinetic constants to enzyme concentrations, allowing for the detailed mechanistic study of proteome allocation.
